# The kinase inhibitor SI113 induces autophagy and synergizes with quinacrine in hindering the growth of human glioblastoma multiforme cells

**DOI:** 10.1186/s13046-019-1212-1

**Published:** 2019-05-17

**Authors:** Silvia Matteoni, Claudia Abbruzzese, Paola Matarrese, Gabriele De Luca, Anna M. Mileo, Stefania Miccadei, Silvia Schenone, Francesca Musumeci, Tobias L. Haas, Giovanni Sette, Carmine M. Carapella, Rosario Amato, Nicola Perrotti, Michele Signore, Marco G. Paggi

**Affiliations:** 1 Section of Cellular Networks and Molecular Therapeutic Targets, Proteomics Unit, IRCCS - Regina Elena National Cancer Institute, Via Elio Chianesi 53, 00144 Rome, Italy; 2 Center for Gender-Specific Medicine, Oncology Unit, Istituto Superiore di Sanità, Rome, Italy; 3 Department of Oncology and Molecular Medicine, Istituto Superiore di Sanità, Rome, Italy; 4 Tumor Immunology and Immunotherapy, IRCCS - Regina Elena National Cancer Institute, Rome, Italy; 5 Department of Pharmacy, University of Genova, Genoa, Italy; 6 Department of General Pathology, Università Cattolica del Sacro Cuore, Rome, Italy; 7 Department of Oncology and Molecular Medicine, Istituto Superiore di Sanità, Rome, Italy; 8 Division of Neurosurgery, IRCCS - Regina Elena National Cancer Institute, Rome, Italy; 9 Department of “Scienze della Salute”, University “Magna Graecia” of Catanzaro, Catanzaro, Italy; 10 RPPA Unit, Proteomics Area, Core Facilities, Istituto Superiore di Sanità, Viale Regina Elena 299, 00162 Rome, Italy

**Keywords:** SI113, Glioblastoma multiforme, RPPA, Autophagy, Quinacrine

## Abstract

**Background:**

Glioblastoma multiforme (GBM), due to its location, aggressiveness, heterogeneity and infiltrative growth, is characterized by an exceptionally dismal clinical outcome. The small molecule SI113, recently identified as a SGK1 inhibitor, has proven to be effective in restraining GBM growth in vitro and in vivo, showing also encouraging results when employed in combination with other antineoplastic drugs or radiotherapy. Our aim was to explore the pharmacological features of SI113 in GBM cells in order to elucidate the pivotal molecular pathways affected by the drug. Such knowledge would be of invaluable help in conceiving a rational offensive toward GBM.

**Methods:**

We employed GBM cell lines, either established or primary (neurospheres), and used a Reverse-Phase Protein Arrays (RPPA) platform to assess the effect of SI113 upon 114 protein factors whose post-translational modifications are associated with activation or repression of specific signal transduction cascades.

**Results:**

SI113 strongly affected the PI3K/mTOR pathway, evoking a pro-survival autophagic response in neurospheres. These results suggested the use of SI113 coupled, for maximum efficiency, with autophagy inhibitors. Indeed, the association of SI113 with an autophagy inhibitor, the antimalarial drug quinacrine, induced a strong synergistic effect in inhibiting GBM growth properties in all the cells tested, including neurospheres.

**Conclusions:**

RPPA clearly identified the molecular pathways influenced by SI113 in GBM cells, highlighting their vulnerability when the drug was administered in association with autophagy inhibitors, providing a strong molecular rationale for testing SI113 in clinical trials in associative GBM therapy.

**Electronic supplementary material:**

The online version of this article (10.1186/s13046-019-1212-1) contains supplementary material, which is available to authorized users.

## Background

Glioblastoma Multiforme (GBM) is the deadliest tumor of the central nervous system and, due to its location, aggressive biological behavior and diffuse infiltrative growth, presents disproportionately high morbidity and mortality. The cornerstone of therapy consists of maximal well-tolerated surgical resection followed by radiotherapy plus concurrent and adjuvant chemotherapy with temozolomide (TMZ) [1]. Despite this optimized treatment schedule, GBM is characterized by high rates of recurrences, therefore the median patient survival ranges from 12 to 15 months, where less than 5% of patients survive for more than 5 years after diagnosis [1]. Moreover, since GBM is characterized by diffusely infiltrative growth and unusual ability in repairing the therapy-induced damages, a complete eradication can be exploited quite rarely [2], which ultimately leads to a high rate of recurrences characterized by increased aggressiveness. Systemic therapy with TMZ, though with its limitations, remains the standard of care for GBM, but the frequent onset of chemoresistance in relapsed GBM generates a compelling need for novel therapeutic strategies. Of note, there is presently no valid alternative to the aforementioned single-drug approach using TMZ in concomitance of radiotherapy or alone in the adjuvant treatment.

SI113, a small molecule identified by virtual screening of a molecular library with respect to SGK1 crystal structures [3], has proven to delay the cell cycle progression with cell accumulation in G0-G1 and block cancer growth in preclinical settings, both in vitro and in vivo [4, 5]. SGK1, a structural and functional analogue of AKT [6], is a key regulator in a number of patho-physiological cell functions [7–9], thus the possibility to modulate its activity can be functional in several diseases [10]. Indeed, SI113 inhibits epithelial-to-mesenchymal transition and subverts cytoskeletal organization in human cancer cells [11] and, specifically in GBM, potentiates the cytotoxic effects of radiotherapy [12] as well as those of mitotic spindle poisons [13]. In the attempt to delve into the mechanism of action of this compound and assess the status of diverse signal transduction pathways in GBM cell lines [14], we initially employed a Reverse-Phase Protein Arrays (RPPA) platform [15–17], a technology designed for multiplexed, antibody-based relative quantification of specific cellular proteins along with their post-translational modifications. RPPA results outlined distinct molecular profiles between anchorage-dependent established cell lines and patient-derived neurospheres as far as pivotal cellular pathways governing cell growth and metabolism are concerned, either at the baseline or under the effect of SI113. Furthermore, SI113 triggered an autophagic response in GBM cells, ultimately leading to cytoprotective autophagy in neurospheres, thus suggesting that its administration concomitant with an inhibitor of the autophagic process could effectively hinder GBM growth.

## Methods

### GBM cell lines

ADF human GBM cells [18] were a gift from Dr. W. Malorni (Istituto Superiore di Sanità, Rome, Italy). U373MG and T98G GBM cells were provided by Dr. C. Leonetti (IRCCS - Regina Elena National Cancer Institute, Rome, Italy). Cell line authentication was performed by short tandem repeat (STR) profiling, which resulted in > = 70% match for 8 loci as per interrogation of the ATCC STR profiling database. Similar to previous reports in the literature [19], our U-373 MG matched ATCC’s U-251 MG cell line. Cells were cultured as previously reported [13], were Mycoplasma-free and used for a maximum of 20 passages.

GBM3-Luc anchorage-independent neurospheres are a primary cell clone, growing in suspension, derived from a specimen of a GBM patient operated in the IRCCS - Regina Elena National Cancer Institute, Rome, Italy, collected according to the current institutional ethical guidelines. GBM3-Luc have been characterized as follows: a) derived from a GBM removed in 2012, negative for MGMT promoter methylation and wild-type for IDH1 gene; b) grow as neurospheres; c) tumorigenic in mice (orthotopic growth); d) CD133: negative; e) CD44: 40% positive; f) CD56: 97% positive; g) engineered to stably express a luciferase reporter gene. GBM-I is another primary neurosphere cell line, gift from Dr. A. Eramo (Istituto Superiore di Sanità, Rome, Italy) [20, 21]. Both neurospheres were cultured in DMEM/F12 stem cell medium as described [20, 21].

### Drugs

SI113 was synthesized as previously reported [3] and diluted at a 50 mM concentration in DMSO. Quinacrine (QC) (Sigma-Aldrich, St. Louis, MO) was diluted at a 10 mM concentration in phosphate-buffered saline (PBS).

### RPPA

For RPPA analysis, 3.5 × 10^3^ GBM3-Luc, ADF and U373MG cells were seeded onto 6-well microtiter plates; then cells from three different passages were used for biological replicates of individual experimental conditions. In details, the RPPA experimental design included two time points, i.e. 2 and 8 h, and two drug doses, namely the concentration resulting in 30 and 50% residual viability as measured at 48 h (see below for details on the cell viability assay used), i.e. IC30 and IC50, respectively. Control samples, i.e. cells treated with a concentration of drug vehicle identical to that of drug-treated samples, were included at each analyzed time point.

Protein extracts as well as RPPA lysate printing and immunostaining, were performed according protocols established in our laboratory [17, 22]. Image analysis for spot recognition, quantification and normalization was carried out using ‘MicroVigene’ v5.2 software (http://www.vigenetech.com/MicroVigene.htm) (VigeneTech Inc). Data analysis was performed on averaged technical and biological replicate values for each individual sample condition by means of ‘R’ v3.5.0 (https://www.r-project.org/) (R Foundation for Statistical Computing) and ‘RStudio’ v1.1.414 https://www.rstudio.com/ (RStudio) using the following installed packages: base, plyr, tidyverse, ggsignif, FactoMineR, factoextra, RColorBrewer, Bioconductor and shiny.

### Immunoblot analysis

In order to validate the RPPA results, cell lysates were processed for western blot as described [23] and filters were probed using the antibodies listed in Additional file 1: Table S1. Further antibodies used throughout the study: mouse anti-p62 (SQSTM1) MoAb GT1478 (1:1000, BD Thermo Fisher Scientific, San José, CA); rabbit anti-LC3 PoAb (1:1000, MBL International, Woburn, MA); mouse anti-β-actin MoAb (1:10000, MP Biomedicals, Aurora, OH); Rabbit anti-Nucleolin PoAb (1:1000, Abcam, London, UK); mouse anti-GAPDH (1:24000, Sigma Aldrich, Saint Louis, MO).

### Cell viability assay

Cells were seeded in 96-well plates at a concentration of 5 × 10^3^ cells/well and then treated with drugs at the given concentrations. CellTiter-Glo Luminescent Cell Viability Assay (Promega, Madison, WI) was employed to determine the relative number of viable cells, after 48 h of treatment, by means of a GLOMAX 96 Microplate Luminometer (Promega). Control samples were treated with the same final concentration of drug solvent(s) (DMSO and/or PBS).

### Colony-forming assay

Anchorage-dependent GBM cells were plated at a concentration of 1–2 × 10^2^ cells/well in 6-well plates. After 24 h, vehicle(s), SI113, QC or a combination of both (as indicated) was added, and the culture was incubated for 48 h. Cells were then washed, cultured for additional 12 d and subsequently stained using a 5% crystal violet solution in order to assess the colony number.

### Neurosphere formation assay

GBM3-Luc and GBM-I cells were plated at 2.5 × 10^5^ cells per well in stem medium with 10 ng/ml bFGF and 20 ng/ml EGF in a 6-well plate and treated with vehicle(s), SI113, QC or a combination of both compounds as indicated. Following 48 h treatment, GBM3-Luc cells were dissociated into single cell suspension by means of TrypLE Express (Gibco, Life Technologies), counted, diluted at the appropriate concentration and re-seeded in triplicate into new 6-well plates (1 × 10^2^ cells per well). At d 26 cells were examined by means of an inverted microscope and neurospheres were counted on averaged triplicates of 9 fields/well using a 4 x objective. Neurosphere counting was blind and independently performed by two investigators.

GBM-I cells were dissociated, diluted and reseeded into new 6-well plates at the concentration of 5 × 10^2^ cells/well. Since GBM-I cells grow more slowly and form smaller neurospheres when compared with GBM3-Luc cells, the former were all pelleted 26 d after treatment, fixed in 2% PFA, stained with 2% crystal violet and cytocentrifuged on a slide.

### Cytofluorimetric assays

Evaluation of autophagy was performed by staining cells with Cyto-ID Autophagy Detection Kit (Enzo Life Sciences, Farmingdale, NY) optimized for detection of autophagy in live cells by flow cytometry [24]. Samples were analyzed with a dual-laser FACScalibur flow cytometer (BD Biosciences Franklin Lakes, NJ).

### Statistical and data analysis

Antibodies were sub-selected based on the concordance between the two analyzed time points. In details, data from all drug treatment conditions were log10-transformed and the Pearson’s correlation indices (‘r’) were calculated, stratified by antibody and cell line, between the 2 and 8 h time points. Endpoints included in subsequent data analyses comprised the roughly 25% displaying r values > 0.64 (third quartile) in at least one cell line out of the three analyzed (Additional file 1: Table S1). AMPK-α pS485 and AMPK-β pS108, but not AMPK-α pT172, scored r values above the threshold criteria. Nonetheless, we included AMPK-α pT172 since we found correlation (r = 0.72) in GBM3-Luc cells between AMPK-α pT172 and its functional substrate ACAC p79. Along similar lines, we utilized 4E-BP1 pT70 and ERK1–2 pT202-pY204, although not reaching the threshold criteria, since they are functionally related to key pathway targets.

Unless otherwise specified i) all experimental conditions were tested in technical triplicates and experiments performed at least three times, ii) all RPPA point-and-line plots include individual values as well as mean ± standard deviation (SD) and iii) all other results are expressed as a mean ± standard error (SE). Data from in vitro experiments were analyzed by One-way ANOVA test followed by Tukey’s Multiple Comparisons Test (GraphPad Prism v5). RPPA results were analyzed by a custom ‘R’ algorithm designed to i) test the normality assumption via Lilliefors or Shapiro-Wilk tests, ii) perform ANOVA and Bonferroni post-hoc tests if the data are normally distributed or iii) perform Kruskal-Wallis followed by Wilcoxon rank sum or signed rank tests as well as by FDR *p* value adjustment, in case of non-normal data. Statistical significance is reported on plots using the following notation: *P < 0.05, **P < 0.01, ***P < 0.001.

The assessment of synergy between two drugs was done using the algorithm described by Fransson et al. [25], where synergy is characterized by a Combination Index (CI) < 0.8, while a CI between 0.8 and 1.2 indicates an additive effect and values > 1.2 indicate an antagonistic effect.

As far as RPPA data are concerned, we opted for *n* = 9 (3 technical × 3 biological replicates) for each individual experimental condition (cell line, drug concentration and time point) in order to achieve a power of 0.8 to detect a statistically significant (FDR 5%) difference in at least 20% of the endpoints, given > 75% non-overlapping populations. We generated custom R code based on ‘R Shiny’ and useful for interactive visualization of RPPA data by plots, dendrograms and heatmaps. The aforementioned code can be made available upon request.

For in vitro data (normally distributed) we tested the homogeneity of variance by Levene’s test and we found out that most of the conditions evaluated were homoscedastic. Accordingly, we performed ANOVA.

## Results

### GBM cell lines, their sensitivity to SI113 and RPPA analysis

Initially, we utilized three human GBM cell lines, two of which were anchorage-dependent, i.e. ADF [18] and U373MG [26], and one (GBM3-Luc), a cell clone isolated from a GBM patient and growing in suspension as neurospheres. In order to characterize anchorage-dependent cells and neurospheres by their pathway-level dependencies, we sought to use RPPA to analyze GBM cells left untreated or treated with SI113. To this end, we selected 114 antibodies, previously pre-validated for use in RPPA, directed against a panel of (phospho-) proteins and protein factors acceptably associated with activation or repression of specific signal transduction cascades. The complete list of the RPPA determinants analyzed is reported in Additional file 1: Table S1.

To evaluate the effects of SI113, we performed a dose-response assay and determined, for individual cell lines, the IC30 and IC50 values after 48 h of SI113 treatment (Table 1). In search for RPPA determinants of early pathway alterations, cells were incubated with SI113, or vehicle (control) for 2 and 8 h.

**Table 1:** IC 30 and IC50 values for SI113 in ADF, U373MG and T98G human GBM anchorage-dependent cell lines and for GBM3-Luc and GBM-I neurospheres

Cell line	IC30 for SI113 (μM)	IC50 for SI113 (μM)
ADF	10.4	19.7
U373MG	7.0	14.45
T98G	5.23	6.65
GBM3-Luc	9.47	19.15
GBM-I	9.10	17.21

Values were determined by titrating the effect of the drug in a cell viability assay after 48 h of treatment. The data reported are the average of two different experiments performed in triplicate

On the one side, and in order to study pathway activation signatures of treated versus untreated anchorage-dependent cells and neurospheres, we performed unsupervised mining on the entire, pre-selected RPPA data subset. On the other side, we compared sample groups by paired statistical tests on a per-antibody basis.

**Fig. 1:**
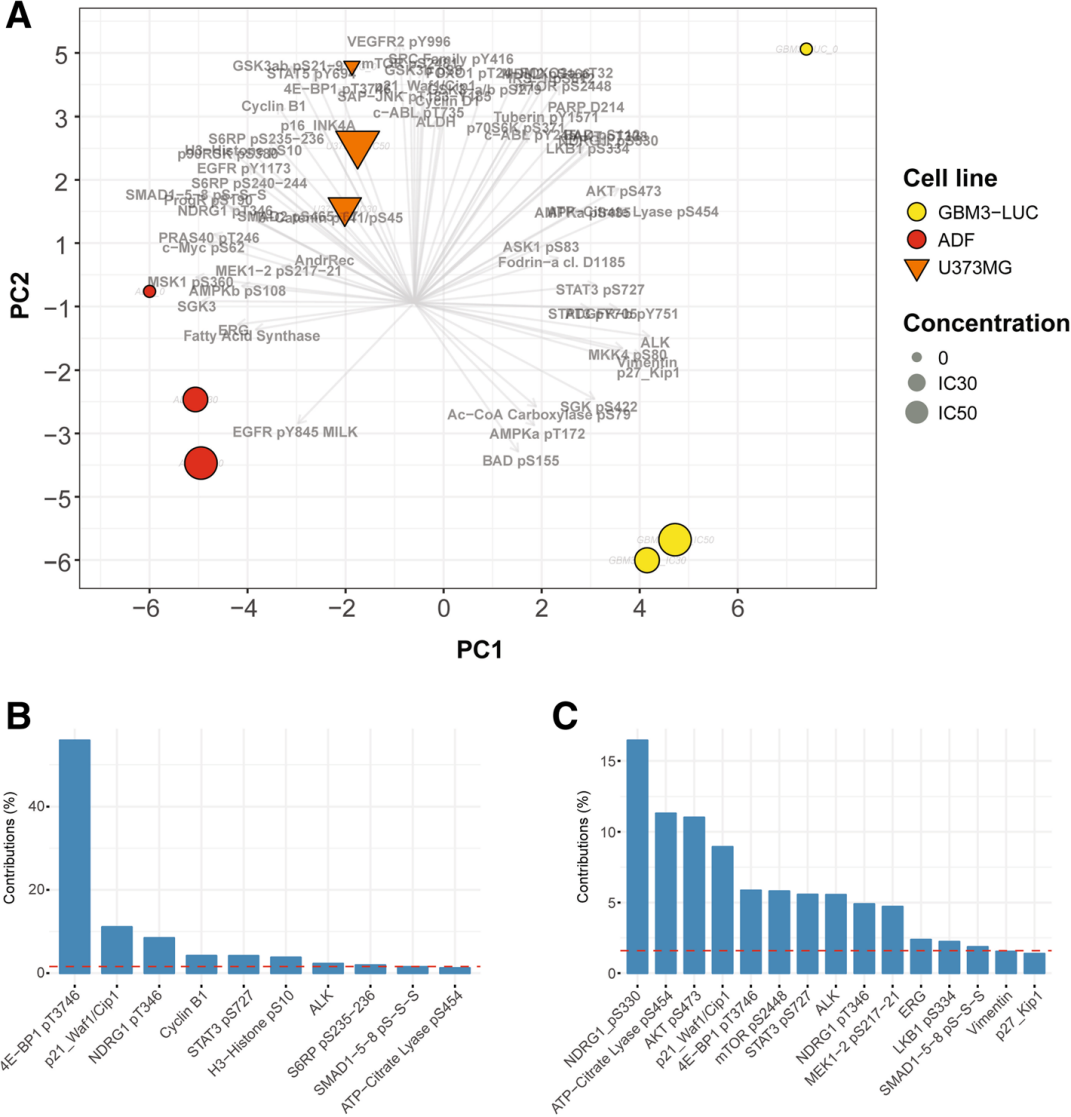
Principal Component Analysis (PCA) of RPPA data. The subset of antibodies selected after thresholding the correlation indices between different time points, was used for PCA on the correlation matrix of data at a single time point (8 h). The bi-plot in (**a**) shows the scores of analyzed samples, i.e. cell lines and SI113 levels, annotated by the color and the size of the symbols, respectively. The relative weight of individual endpoints (loadings) is overlaid and highlighted by arrows. Histograms in (**b**) and (**c**) show the relative contribution of endpoints (expressed as percentage of the contribution of all variables) with significantly high correlations in the first and the second PC, respectively. The red, dashed line indicates the threshold level of relative contribution

Principal component analysis (PCA) showed that, regardless of the treatment with SI113, GBM3-Luc cells demonstrated different, distinctive signaling equilibria, mainly characterized by key actors of cell cycle pathway (PC1, Fig. 1a and b). Intriguingly, while RPPA expression of anchorage-dependent cells was not dramatically affected by SI113 treatment, in neurospheres the drug induced sizeable changes of RPPA endpoints involved in cell growth, proliferation and metabolism (PC2, Fig. 1a and c).

In-depth comparison of baseline and treated samples in anchorage-dependent cells and neurospheres confirmed the presence of individual, non-overlapping molecular responses to SI113, conveyed by a defined panel of pathway players.

#### PI3K/mTOR axis

**Fig. 2:**
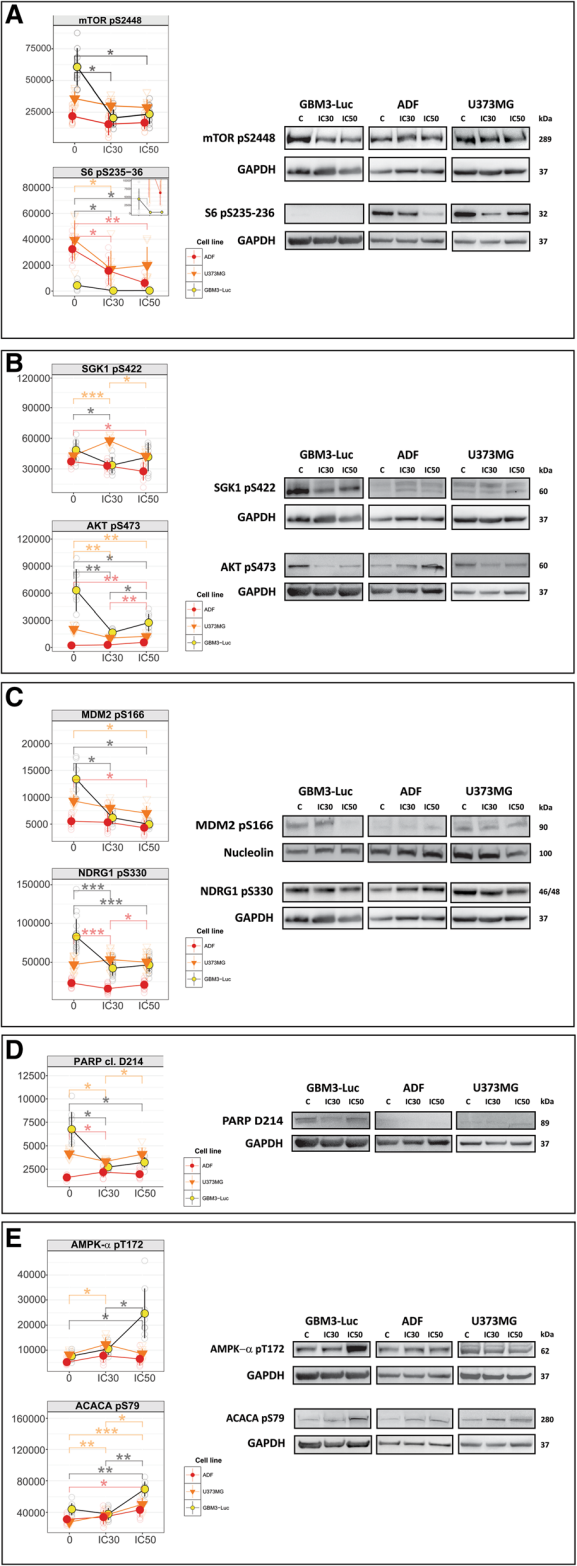
RPPA analysis of mTORC1 and mTORC2 activity. GBM3-Luc, ADF and U373MG cells were incubated in the absence or in the presence of SI113 and then processed for RPPA and Western blot (left and right panels, respectively). SI113 was employed at the IC30 and IC50 concentrations specific for each cell line. Unless otherwise specified all RPPA plots refer to results obtained at 8 h of exposure to SI113. When significant, statistical comparisons (Wilcoxon signed-rank test, FDR-adjusted *p* values) are reported on each individual plot. Statistical significance coding is described in the Materials and Methods section of the manuscript. **a**. mTORC1. RPPA plots represent the trend of mTOR pS2448 and S6 pS235–36 phosphorylation as readouts of mTORC1 activation status. mTOR pS2448 and S6 pS235–36 normalization was performed by GAPDH quantification. **b**. mTORC2. Representative RPPA plots of SGK1 pS422 (2 h time point shown) and AKT pS473 show the trend of mTORC2 activity upon treatment with SI113. SGK1 pS422 was normalized against the GAPDH determination previously used for mTOR pS2448, while AKT pS473 in U373MG cells shares the loading control (GAPDH) with S6 pS235–36. **c.** AKT and SGK1 activity. RPPA plots represent the phosphorylation trend of MDM2 and NDRG1, which are targets of the AKT/SGK1 activity, under the effect of SI113. Nucleolin content was used for MDM2 pS166 normalization while GAPDH, the same as the one reported for SGK1 pS422 normalization in panel B, was used for NDRG1 pS330 normalization. **d**. Apoptosis. RPPA plots display the trend of cleaved PARP (D214) after SI113 treatment. GAPDH determination used for PARP D214 normalization in GBM3-Luc and ADF cells was done on the same filter used for AKT pS473. **e.** Autophagy. Plots of ACACA pS79 and AMPK-α pT172 RPPA levels are shown here to represent the trend of the autophagic process under the effect of SI113. ACACA pS79 in U373MG cells share the same GAPDH normalization used for AKT pS473 and S6 pS235–236. AMPK-α pT172 in GBM3-Luc and ADF cells share the same GAPDH normalization used for S6 pS235–236.kDa = apparent molecular mass

SI113 treatment caused a significant decrease of mTOR pS2448 (Fig. 2a) and its downstream target 4E-BP1 (i.e. 4E-BP1 pT37–46 and 4E-BP1 pT70) in neurospheres, but not in anchorage-dependent cells (Fig. 2a and Additional file 2: Figure S1 respectively). Therefore we focused also on another critical mTORC1 target, i.e. S6 pS235–36, and found that SI113 affected its levels in all cell lines analyzed (Fig. 2a). The effects of SI113 on mTOR pS2448 and S6 pS235–36 status were evident already at the IC30 dose. Since the phosphorylation of both factors is a readout of the mTORC1 complex activity [6, 27], our data suggest that SI113 targeted, either directly or indirectly, the PI3K/mTOR axis. Of note, basal mTOR pS2448 resulted elevated in neurospheres and lower, and fairly comparable, in anchorage-dependent cells, while baseline S6 pS235–236 displayed an opposite pattern, being particularly low in neurospheres and increased in anchorage-dependent cells. Nonetheless, the decrease of S6 pS235–236 following SI113 treatment remained appreciable in neurospheres (Fig. 2a left, inset).

To assess the effects of SI113 on the mTORC2 complex, we examined mTOR pS2481 [27] as well as AKT pS473 [27] and SGK1 pS422 [28], the latter two being known substrates of the mTORC2 kinase activity. Indeed, SI113 appreciably down-regulated mTOR pS2481 in neurospheres but not in anchorage-dependent cells (Additional file 2: Figure S1). These results were paralleled by a significant reduction of AKT pS473 and SGK1 pS422 after treatment with the lowest dose of SI113 in GBM3-Luc cells only (Fig. 2b, left).

In order to achieve a full comprehension of SI113-mediated changes in the PI3K/mTOR pathway, we selected two key readouts, i.e. MDM2 and NDRG1 [6], which are phosphorylated by AKT and SGK1 in serine 166 [29, 30] and 330 [31–33], respectively. In the presence of SI113, both factors resulted considerably hypo-phosphorylated in neurospheres. Vice versa, in anchorage-dependent cells only the highest dose of SI113 caused a significant decrease of MDM2 pS166 and only one out of the two anchorage-dependent cells analyzed underwent a reduction in NDRG1 pS330 at SI113 IC30 (Fig. 2c, left). Interestingly, RPPA levels of FOXO1 pS256 and FOXO1 pT24-FOXO3a pT32 were selectively affected by SI113 in neurospheres mirroring, in such cells, the drug-induced decline of AKT phosphorylation and, to a lesser extent, that of SGK1, being both AKT and SGK1 upstream regulators of FOXOs’ activity (Additional file 2: Figure S1) [34–37].

#### Apoptosis

SI113 has been shown to trigger apoptosis in cultured cancer cells [4, 5]. Thus we measured the amount of cleaved PARP (PARP D214) [38] after treatment with SI113. Apparently, PARP D214 displayed a sizeable, significant SI113-dependent reduction mainly in neurospheres (Fig. 2d, left).

Altogether, the RPPA output obtained after exposure of GBM cells to SI113 showed i) an overall reduction of mTORC1 activity paralleled by ii) a neurosphere-selective mTORC2 down-modulation accompanied by iii) a remarkable decrease in AKT pS473 and SGK1 pS422, mostly in neurospheres, and iv) a reduction of apoptosis-related endpoints. These results suggest that neurospheres, under the effect of SI113, might co-opt for a survival-oriented autophagic process.

#### Autophagy

Intrigued by the above results, we investigated RPPA endpoints involved in the autophagy pathway. Indeed, the neurosphere-specific inhibition of mTOR pS2448 by SI113 was mirrored by a drug-induced increase in phosphorylation of the energy sensor AMPK-α (at threonine 172) (AMPK-α pT172 a.k.a. AMPK-α pT183) and its substrate ACACA pS79 (a.k.a. ACACA pS80), two specific readouts of the onset of an autophagic process [39, 40] (Fig. 2e, left). Indeed, while AMPK-α phosphorylation correlates with a raise in its kinase activity, AMPK-α-mediated phosphorylation of ACACA at S79 results in inactivation of the ACACA enzyme activity. Therefore, we hypothesized that triggering of an autophagic process would result in a raise of NADPH levels with concomitant down-modulation of fatty-acid synthesis and increased rates of fatty-acid oxidation [41, 42]. All the RPPA results were validated by western blotting (Fig. 2a, b, c, d and e, right**)**.

In line with such a molecular scenario, we found a neurosphere-specific down-regulation of fatty-acid synthase (FASN) after SI113 treatment (Additional file 2: Figure S1), indicating the selective stimulation of an energy-saving, pro-survival autophagic process in neurospheres.

Interestingly, mTOR pS2448 and ACACA pS79 showed significant SI113-mediated changes and these molecular readouts of autophagy were found pronounced in neurospheres as compared to anchorage-dependent cells. Indeed, although SI113 inhibited ACACA pS79 in a dose-dependent manner in all analyzed cell lines, in GBM3-Luc cells the drug selectively induced a concomitant increase of AMPK-α pT172 and decrease of mTOR pS2448. Moreover, despite a SI113-induced decline in Cyclin B1 in all analyzed cell lines, the levels of phosphorylated ERKs (ERK1–2 pT202-pY204) underwent significant reduction in neurospheres only (Additional file 2: Figure S1). Therefore, it could be argued that these cells halted their proliferation state and, differently from anchorage-dependent lines, were able to mount an autophagic response. In line with such a hypothesis, we found that neurospheres and anchorage-dependent cells displayed differential ground level phosphorylation of S6 pS235–236, a bona fide readout of the autophagy regulator mTORC1 [43].

Altogether, our RPPA data suggest, as previously demonstrated in other model systems [12, 13], that SI113 elicited an autophagic response in GBM cells particularly evident in neurospheres and arguably associated with a pro-survival, autophagic strategy utilized to endure the treatment with SI113.

The raw data concerning the RPPA platform output are listed in Additional file 3: Table S2.

### Synergy between SI113 and the antimalarial drug quinacrine in restraining GBM cell growth

Recently, antimalarial drugs belonging to the 4-amino quinoline class have been demonstrated to inhibit autophagy at its late stages [44] contributing to hamper cancer cell growth, mainly in the subset of tumor-initiating cells [45–48]. We therefore employed in our experimental setting the quinolone derivative quinacrine (QC) [49], due to its highest activity in inhibiting autophagy in glioma cells and ability to cross the Blood-Brain Barrier [44]. Thus, the effect of SI113 and QC, as well as that of their combined administration, was assayed in GBM cells, where QC was envisaged to impair the autophagic survival process elicited by SI113. Sensitivity to QC was assessed for the GBM cell lines ADF, U373MG and GBM3-Luc neurospheres. We also assayed the T98G anchorage-dependent GBM cell line [32] and the GBM-I neurospheres [20, 21]. IC50 values for QC are reported in Table 2. All these cells appeared susceptible to QC in the low micro-molar range, thus confirming the ability of quinoline-derived compounds to hinder per se GBM growth [50–52].

**Table 2:** IC50 values for QC in ADF, U373MG and T98G human GBM anchorage-dependent cell lines and for GBM3-Luc and GBM-I neurospheres

Cell line	IC50 for QC (μM)
ADF	2.46
U373MG	2.39
T98G	2.18
GBM3-Luc	4.79
GBM-I	5.43

Values were determined by titrating the effect of the drug in a cell viability assay after 48 h of treatment. The data reported are the average of two different experiments performed in triplicate

#### Cell viability assay

**Fig. 3:**
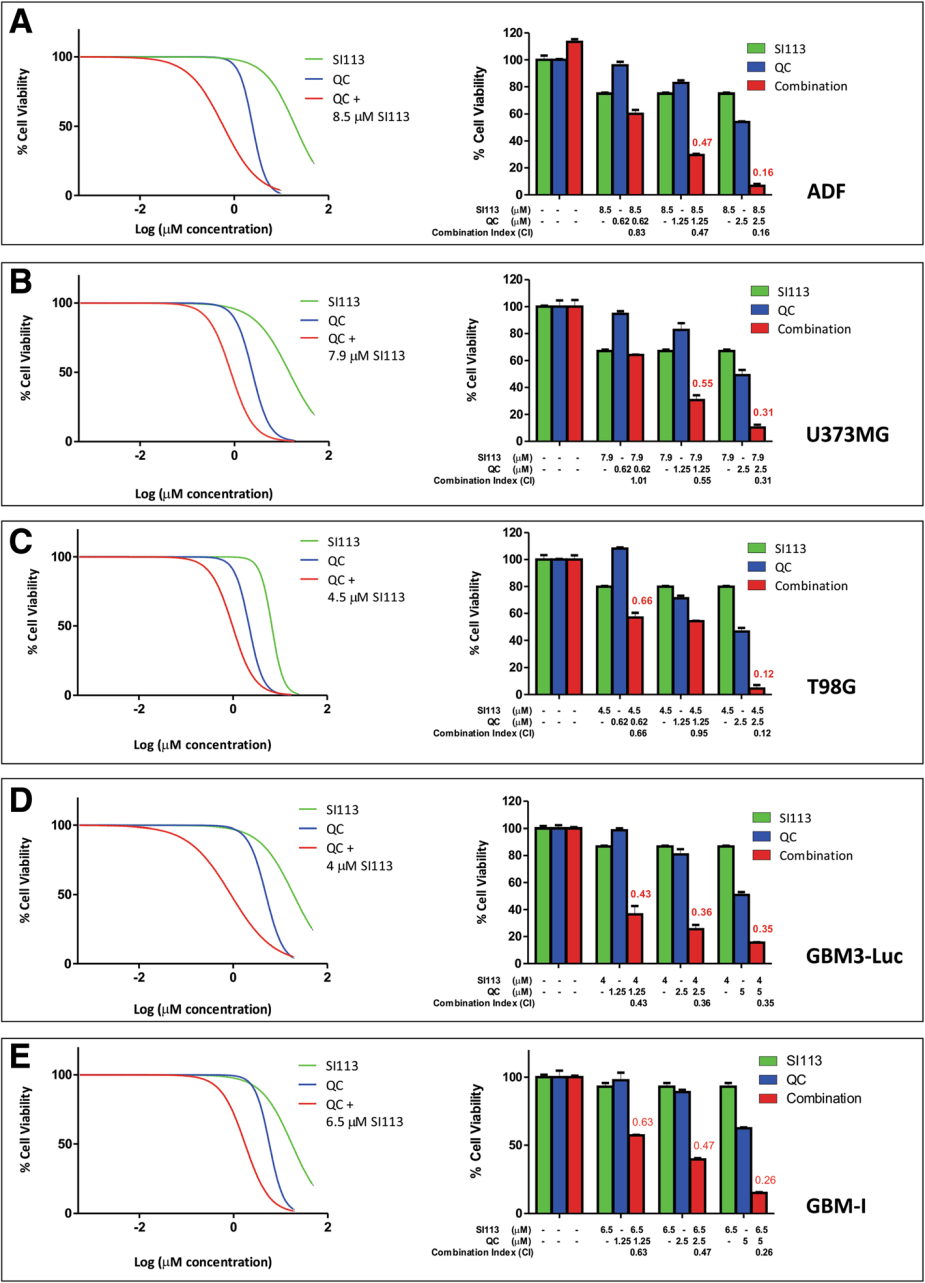
Cell Viability Assay. Dose-response effects of SI113 (green), QC (blue) and QC plus a constant (k) SI113 concentration (red) on percent cell viability (left panels) and histograms showing cell viability at selected drug concentrations, as indicated, to highlight the effect of the association of the two drugs (right panels). The effects of QC and SI113 combination drugs were considered synergistic when the CI was < 0.8 (in red). SI113 k values were 8.5, 7.9, 4.5, 4 and 6.5 μM for ADF (**a**), U373MG (**b**), T98G (**c**), GBM3-Luc (**d**) and GBM-I cells (**e**), respectively

To highlight a potential synergistic effect between the two drugs, SI113 was employed at a fixed concentration, varying among the different cell lines according to their susceptibility to the drug, as follows: 8.5 μM for ADF; 7.9 μM for U373MG; 4.5 μM for T98G; 4 μM for GBM3-Luc and 6.5 μM for GBM-I. To assay for a synergistic effect, increasing doses of QC were then employed, ranging from 0.078 to 20 μM. Cell viability was assessed after 48 h. By using the algorithm described by Fransson et al. [25], we analyzed the outcome of the drug combination compared with that of the compounds used as single agents (Fig. 3a, b, c, d and e, left). Indeed, the addition of QC to SI113 yielded a decrease in cell viability definitely attributable to a synergistic effect of the two drugs within an extended range of QC concentrations for all the cell lines. (Fig. 3a, b, c, d and e, right).

#### Clonogenic assays

**Fig. 4:**
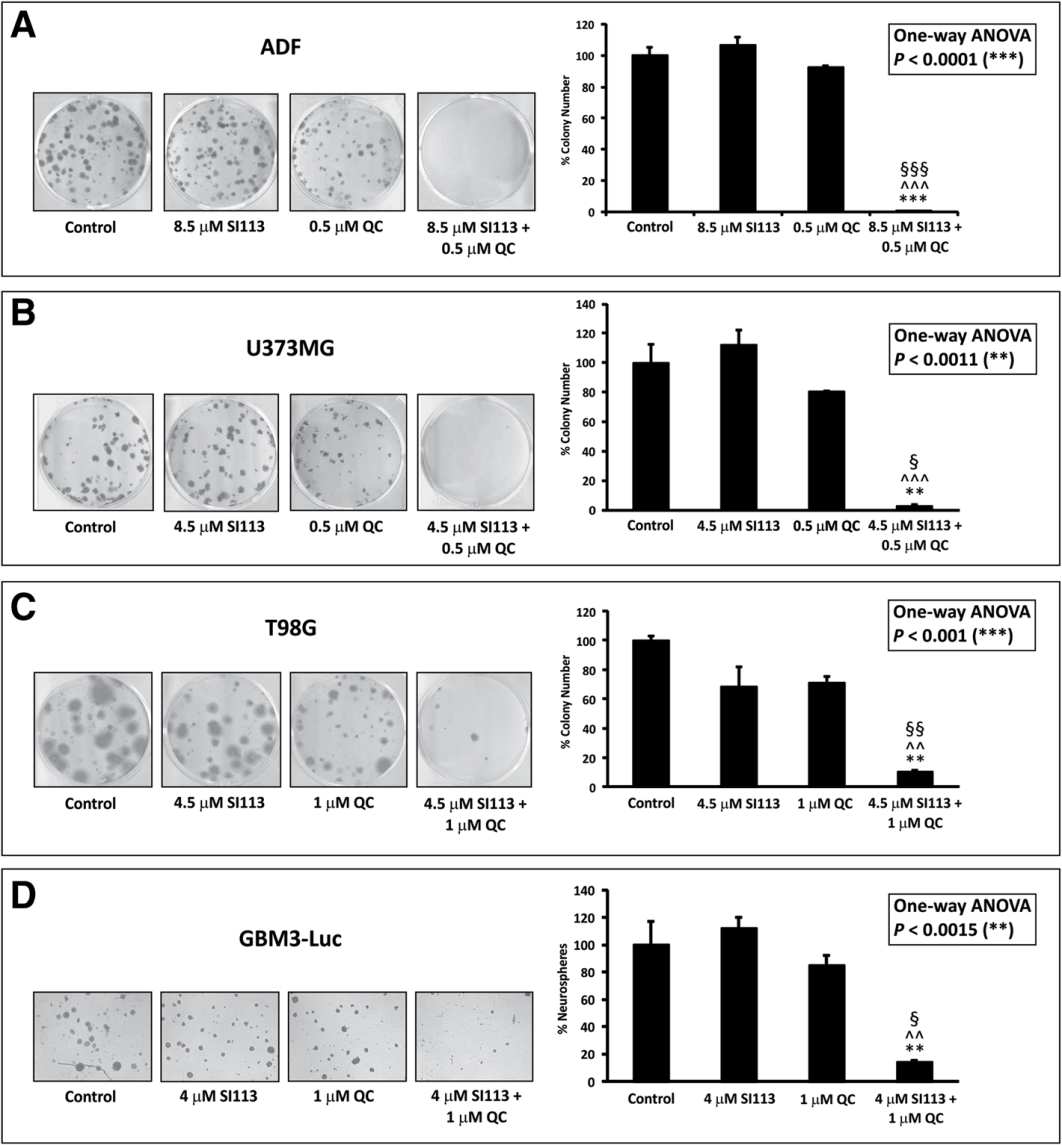
Clonogenic Assay. **a**. ADF cells were exposed to solvent(s) (Control), 8.5 μM SI113, 0.5 μM QC or their association for 48 h and then allowed to grow and form colonies for the subsequent 12 d. Cell colonies, after staining with crystal violet (left), were counted and the values reported as percent colony number in the histogram (right). **b**. U373MG cells treated as in panel a, except for the use of SI113 at a concentration of 4.5 μM. **c.** T98G cells treated as in panel b, except for the use of QC at a concentration of 1 μM. **d.** GBM3-Luc cells were exposed to solvent(s) (Control), 4 μM SI113, 1 μM QC or their association for 48 h and then allowed to grow and form colonies for the subsequent 26 d. Cell colonies were observed and photographed in phase contrast microscopy (left), counted and the values reported as percent colony number in the histogram (right). In these panels, statistical analysis among groups was done using the One-way ANOVA test followed by the Tukey’s Multiple Comparison Test (* significance vs. Control; ^ significance vs. SI113; § significance vs. QC)

In this setup, drug doses were modified as indicated in Fig. 4.

##### Colony-forming assay

ADF, U373MG and T98G cells were exposed to the drug(s) or their respective solvent(s) and the effect of the treatment was evaluated via the inhibition of the number of cell colony formation. For all these cell lines, cloning efficiency was dramatically reduced when SI113 and QC were used in combination (Fig. 4a, b and c, pictures on the left and histograms on the right).

##### Neurosphere formation assay

In the case of GBM3-Luc neurospheres we adopted a different technique. As shown in Fig. 4d (pictures on the left and histograms on the right), a potentiation of the effects between SI113 and QC in restraining neurosphere formation was clearly evident also in this cell line. Statistical significances for each panel of Fig. 4 are reported in the Figure legend. Growth and morphological characteristics of the GBM-I neurospheres did not allow a reliable count of the colonies in dishes; in this case, cells needed to be cytocentifuged after the treatments. In GBM-I cells, the growth restraining effect of SI113 and QC was evident when administered in combination, while SI113 alone appeared to stimulate the formation of colonies **(**Additional file 4: Figure S2**)**.

### Analysis of the autophagic process in GBM cells after exposure to SI113 and/or QC

For a precise analysis of the autophagic processes, the specific determinants LC3-I/LC3-II and p62 [53] were assayed by western blot after exposure to SI113 and/or QC at the doses indicated above in the clonogenic assay for 48 h and compared to controls.

All three anchorage-dependent GBM cell lines, after exposure to SI113, underwent an increase in the amount of both LC3 II and p62, possibly related to a block of the autophagic flux or induction of cytotoxic autophagy [53]. As expected, the autophagy blocker QC induced an increase in the amount of p62 in U373MG and T98G cells, which was not the case in ADF cells. When SI113 and QC were administered together, there was a further increase in the amount of p62 in all the three cell lines.

When autophagy was analyzed in neurospheres, GBM3-Luc cells showed, in the presence of SI113, a relative increase in the LC3 II band, while in GBM-I cells a visible increase was apparent along with a decrease in the amount of LC3 I. Under the same conditions, a decrease in the amount of p62 was appreciable under the effect of SI113. These scenarios are all compatible with an increased autophagic rate [53]. In the presence of QC, a noticeable block of the autophagic process was evident in both cell lines, as assumed by the coupled increase of LC3 II and p62 [53]. Interestingly, where the cytotoxic effects of the association of SI113 with QC was more evident, a dramatic increase in LC3 II was observed in concomitance with an increase of p62. A noticeable decrease in β-actin amount, here used as a loading control, was also apparent, a phenomenon attributed to intracellular degradation events associated with cell death.

**Fig. 5:**
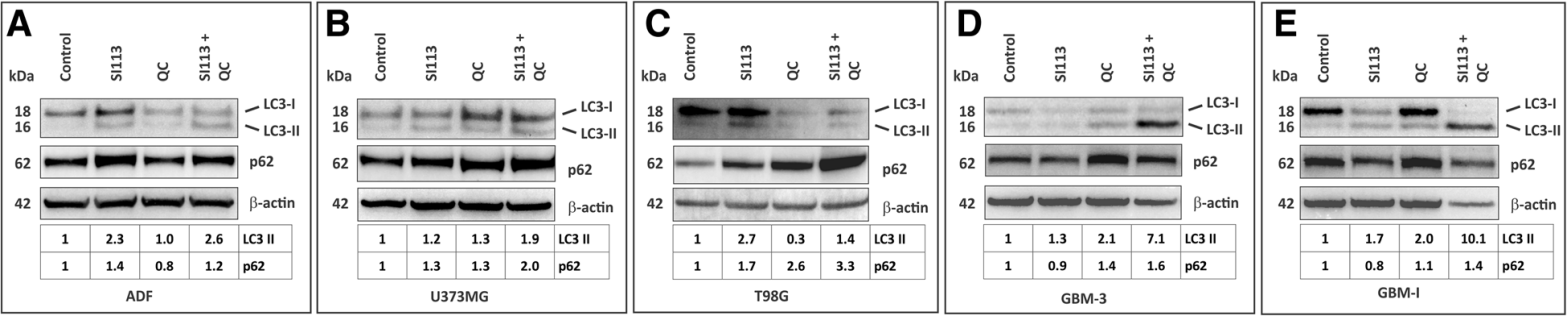
Analysis of the autophagic process. ADF, U373MG, T98G, GBM3-Luc and GBM-I (Panels **a** to **e**) GBM cells were incubated with SI113, QC or their combination using the drug doses employed in Fig. 4 for 48 h and then assayed via western blot for LC3 and p62, as determinants of the autophagic process. Semi-quantitative assessment of LC3 II and p62 amounts, normalized against the β-actin content, is reported for each cell line. kDa = apparent molecular mass

Semi-quantitative assessment of LC3 II and p62 amounts, normalized against the β-actin content, is reported for each cell line (Fig. 5a, b, c, d and e). These results were also validated by flow cytometry using the CYTO-ID autophagy detection kit (Table 3).

**Table 3:** Cytofluorimetric determination of autophagy in GBM cells exposed to SI113 and/or QC

Cell Line	Control	SI113	QC	SI113 + QC
ADF	60.3 ± 4.2	68.9 ± 4.7	82.3 ± 7.2	99.7 ± 7.1
U373MG	75.4 ± 7.7	82.7 ± 5.4	87.6 ± 5.7	95.4 ± 6.5
T98G	73.3 ± 6.5	92.7 ± 4.4	86.5 ± 4.1	95.4 ± 9.3
GBM3-Luc	59.4 ± 5.6	64.5 ± 5.4	71.9 ± 5.1	94.6 ± 6.7
GBM-I	20.9 ± 2.2	25.8 ± 2.5	28.5 ± 2.1	36.8 ± 2.9

Cytofluorimetric assay for autophagy (see Materials and Methods) in GBM cell lines that were exposed to the drugs as specified in Fig. 4. Autophagic process was quantified by analyzing the median fluorescence intensity values and is expressed as Arbitrary Units

## Discussion

The small molecule SI113 was originally identified as an inhibitor of the SGK1 kinase activity and the role of the drug in this context has been documented satisfactorily [4, 5, 12, 13]. SGK1 plays a key functional role in the PI3K/mTOR pathway and is able to sustain AKT-independent mTORC1 activation [6].

The strong impact of SI113 on cancer cell survival has been highlighted by several previous studies. Nonetheless, here we aimed at exploring in depth the pharmacological capability of SI113 in interfering with major signal transduction pathways in GBM cells. Indeed, SI113 was effective in hindering proliferation and cloning efficiency in anchorage-dependent GBM cell lines and neurospheres.

RPPA revealed a peculiar role of SI113 in GBM3-Luc neurospheres, where it provoked a rapid (already at 2 h and IC30) reduction of cell cycle related endpoints, i.e. Cyclin B1 and phospho-ERKs, and promoted a survival-oriented autophagic process. Indeed, upon treatment with SI113, a series of key pathway players, including mTOR pS2448 and its downstream targets 4E-BP1 pT37–46 and S6 pS235–236, as well as AMPK-α pT172 and ACACA pS79, displayed a striking correlation selectively in neurospheres. Concordantly, treatment with SI113 caused a decrease in cleaved PARP D214 in GBM3-Luc cells, thus excluding the involvement of an apoptotic process in these cells. In line with these data, analysis of the expression of LC3 and p62 showed that both GBM-3 Luc and GBM-I neurospheres underwent autophagy when exposed to SI113.

It should be remarked that GBM is characterized by the concomitance of diverse pathway alterations including, but not limited to, RTKs, PI3K/mTOR, Ras/MAPKs and cell cycle [54]. In this regard, it is widely accepted that mTOR inhibition leads to ERK activation in a PI3K-dependent manner [55] and that receptor tyrosine kinase-driven signals are rewired accordingly to selective blockade of either PI3K/mTOR or Ras/MAPK axes [56, 57]. These acquired notions pose the rationale for designing combination therapies involving concomitant targeting of PI3K and MAPKs [58]. Interestingly, SI113 was capable of targeting neurospheres by inhibition not only of PI3K/mTOR pathway but also of the MAPKs cascade, as represented by the noticeable drop in the levels of ERK1–2 phosphorylation in SI113-exposed GBM3-Luc cells (Additional file 4: Figure S2). Such a scenario implies that, for a minimally effective inhibition of GBM cell growth, both the aforementioned pathways should be targeted [59]. Once again, SI113 is a very interesting candidate compound for GBM therapy, being capable of hindering multiple pathways and thus exposing GBM3-Luc neurospheres vulnerabilities.

Additionally, the autophagy inhibitor QC, which, as expected [50–52], displayed considerable toxicity towards all the GBM cell lines tested (see Table 1) showing also an unquestionable ability to cooperate with SI113 in hindering GBM cells growth capabilities. Indeed, we found a strong synergy between SI113 and QC in contrasting key features of both anchorage-dependent GBM cells and neurospheres. Since the latter ones are usually resistant to standard anticancer treatments and display cancer stem cells behaviors, our results hold a substantial clinical significance.

Recently, ferroptosis, a peculiar form of cell death occurring through Fe (II)-dependent lipid peroxidation, has been shown to play a major role in cancer cell apoptotic processes [60]. In addition, it has been reported that the blockade of autophagy by means of QC can enhance sensitivity to TMZ in GBM neurospheres by igniting ferroptosis [61].

In summary, autophagy allows GBM cells to survive in a hostile environment and is regarded as a cytoprotective adaptive reaction, particularly in cancer stem cells [62–64]. Herein, exposure to SI113 forced neurosphere cells towards a pro-survival autophagic response that was efficiently constrained by the autophagy inhibitor QC, thus provoking the suppression of a survival pathway that these cells likely depend on. Therefore, we envisage a combination therapy approach that, differently from standard “one-hit” targeted therapies, could be based on synthetic lethality, inhibiting primitive cancer cell survival pathways, being thus less prone to the emergence of resistant clones and providing strong benefits in a foreseen clinical setting. Indeed, GBM due to its heterogeneity cannot be considered as a “single pathway disease” and doesn’t represent the best environment to assay the effects of a targeted therapy. Conversely, in this disease there is a window of opportunity for the so-called dirty drugs that, far from being specific, can take advantage of selective vulnerabilities of GBM cells, e.g. dependence from aberrant energy supply pathways or pro-survival autophagic response to hostile environment. In such a scenario, our results show the importance to have a complete signal transduction portrait in order to address the most efficient therapeutic strategies.

## Conclusions

All so far published in vitro and in vivo preclinical studies based on SI113 hold great promises for its use in a combinatorial therapeutic regimen in cancer, including GBM [4, 12]. In this respect, a novel therapeutic strategy consisting in TMZ plus SI113 and QC, may provide an effective drug cocktail against GBM [14]. Notably, SI113 has been demonstrated to potentiate the effects of radiotherapy in GBM cells [12], thus advocating its clinical use in GBM. Furthermore, QC or other quinolone derivatives, e.g. the widely used chloroquine, display well-established levels of toxicity and dosage in humans, making them ideal candidates for drug repositioning [14].

Therefore, all the aforementioned elements converge in supporting a Phase 1 clinical trial for safety and dosage assessment of SI113 and QC or its analogs, in order to evaluate the effects of their co-administration with TMZ (and, when still possible, radiation therapy) in GBM patients who have exhausted all available treatment options.

## Additional files

Additional file 1: **Table S1.** Complete list of antibodies used for RPPA analysis and their main related information. (PDF 26 kb)

Additional file 2: **Figure S1.** Additional RPPA endpoints. (PDF 89474 kb)

Additional file 3: **Table S2.** Raw RPPA data. (XLSX 126 kb)

Additional file 4: **Figure S2.** Clonogenic Assay. (PDF 440 kb)

## Abbreviations

CI: Combination index
GBM: Glioblastoma multiforme
PBS: Phosphate-buffered saline
PCA: Principal component analysis
QC: Quinacrine
RPPA: Reverse-Phase Protein Arrays
SD: Standard deviation
SE: Standard error
STR: Short tandem repeat
TMZ: Temozolomide

## References

[1] Stupp R, Mason WP, van den Bent MJ, Weller M, Fisher B, Taphoorn MJ (2005). Radiotherapy plus concomitant and adjuvant temozolomide for glioblastoma. N Engl J Med.

[2] Osswald M, Jung E, Sahm F, Solecki G, Venkataramani V, Blaes J (2015). Brain tumour cells interconnect to a functional and resistant network. Nature.

[3] Ortuso F, Amato R, Artese A, D'Antona L, Costa G, Talarico C (2014). In silico identification and biological evaluation of novel selective serum/glucocorticoid-inducible kinase 1 inhibitors based on the pyrazolo-pyrimidine scaffold. J Chem Inf Model.

[4] D'Antona L, Amato R, Talarico C, Ortuso F, Menniti M, Dattilo V (2015). SI113, a specific inhibitor of the Sgk1 kinase activity that counteracts cancer cell proliferation. Cell Physiol Biochem.

[5] Talarico C, D'Antona L, Scumaci D, Barone A, Gigliotti F, Fiumara CV (2015). Preclinical model in HCC: the SGK1 kinase inhibitor SI113 blocks tumor progression in vitro and in vivo and synergizes with radiotherapy. Oncotarget..

[6] Castel P, Ellis H, Bago R, Toska E, Razavi P, Carmona FJ (2016). PDK1-SGK1 signaling sustains AKT-independent mTORC1 activation and confers resistance to PI3Kalpha inhibition. Cancer Cell.

[7] Dattilo V, D'Antona L, Talarico C, Capula M, Catalogna G, Iuliano R (2017). SGK1 affects RAN/RANBP1/RANGAP1 via SP1 to play a critical role in pre-miRNA nuclear export: a new route of epigenomic regulation. Sci Rep.

[8] Spagnuolo R, Dattilo V, D'Antona L, Cosco C, Tallerico R, Ventura V, et al. Deregulation of SGK1 in ulcerative colitis: a paradoxical relationship between immune cells and colonic epithelial cells. Inflamm Bowel Dis. 2018. 10.1093/ibd/izy158. 10.1093/ibd/izy158 29788407

[9] Abbruzzese C, Mattarocci S, Pizzuti L, Mileo AM, Visca P, Antoniani B (2012). Determination of SGK1 mRNA in non-small cell lung cancer samples underlines high expression in squamous cell carcinomas. J Exp Clin Cancer Res.

[10] Lang F, Voelkl J (2013). Therapeutic potential of serum and glucocorticoid inducible kinase inhibition. Expert Opin Investig Drugs.

[11] Abbruzzese C, Matteoni S, Persico M, Ascione B, Schenone S, Musumeci F, et al. The small molecule SI113 hinders epithelial-to-mesenchymal transition and subverts cytoskeletal organization in human cancer cells. J Cell Physiol. 2019;Accepted for publication. 10.1002/jcp.28816. 10.1002/jcp.28816 31099037

[12] Talarico C, Dattilo V, D'Antona L, Barone A, Amodio N, Belviso S (2016). SI113, a SGK1 inhibitor, potentiates the effects of radiotherapy, modulates the response to oxidative stress and induces cytotoxic autophagy in human glioblastoma multiforme cells. Oncotarget.

[13] Abbruzzese C, Catalogna G, Gallo E, di Martino S, Mileo AM, Carosi M (2017). The small molecule SI113 synergizes with mitotic spindle poisons in arresting the growth of human glioblastoma multiforme. Oncotarget..

[14] Abbruzzese C, Matteoni S, Signore M, Cardone L, Nath K, Glickson JD (2017). Drug repurposing for the treatment of glioblastoma multiforme. J Exp Clin Cancer Res.

[15] Pierobon M, Wulfkuhle J, Liotta L, Petricoin E (2015). Application of molecular technologies for phosphoproteomic analysis of clinical samples. Oncogene..

[16] Pierobon M, Vanmeter AJ, Moroni N, Galdi F, Petricoin EF (2012). Reverse-phase protein microarrays. Methods Mol Biol.

[17] Manic G, Signore M, Sistigu A, Russo G, Corradi F, Siteni S (2018). CHK1-targeted therapy to deplete DNA replication-stressed, p53-deficient, hyperdiploid colorectal cancer stem cells. Gut..

[18] Malorni W, Rainaldi G, Rivabene R, Santini MT (1994). Different susceptibilities to cell death induced by t-butylhydroperoxide could depend upon cell histotype-associated growth features. Cell Biol Toxicol.

[19] Ishii N, Maier D, Merlo A, Tada M, Sawamura Y, Diserens AC (1999). Frequent co-alterations of TP53, p16/CDKN2A, p14ARF, PTEN tumor suppressor genes in human glioma cell lines. Brain Pathol.

[20] Vellanki SH, Grabrucker A, Liebau S, Proepper C, Eramo A, Braun V (2009). Small-molecule XIAP inhibitors enhance gamma-irradiation-induced apoptosis in glioblastoma. Neoplasia.

[21] Eramo A, Ricci-Vitiani L, Zeuner A, Pallini R, Lotti F, Sette G (2006). Chemotherapy resistance of glioblastoma stem cells. Cell Death Differ.

[22] Marziali G, Signore M, Buccarelli M, Grande S, Palma A, Biffoni M (2016). Metabolic/proteomic signature defines two glioblastoma subtypes with different clinical outcome. Sci Rep.

[23] Amato R, Scumaci D, D'Antona L, Iuliano R, Menniti M, Di Sanzo M (2013). Sgk1 enhances RANBP1 transcript levels and decreases taxol sensitivity in RKO colon carcinoma cells. Oncogene..

[24] Mizushima N, Yoshimori T, Levine B (2010). Methods in mammalian autophagy research. Cell..

[25] Fransson A, Glaessgen D, Alfredsson J, Wiman KG, Bajalica-Lagercrantz S, Mohell N (2016). Strong synergy with APR-246 and DNA-damaging drugs in primary cancer cells from patients with TP53 mutant high-grade serous ovarian cancer. J Ovarian Res.

[26] Garufi A, Trisciuoglio D, Porru M, Leonetti C, Stoppacciaro A, D'Orazi V (2013). A fluorescent curcumin-based Zn (II)-complex reactivates mutant (R175H and R273H) p53 in cancer cells. J Exp Clin Cancer Res.

[27] Copp J, Manning G, Hunter T (2009). TORC-specific phosphorylation of mammalian target of rapamycin (mTOR): phospho-Ser2481 is a marker for intact mTOR signaling complex 2. Cancer Res.

[28] Garcia-Martinez JM, Alessi DR (2008). mTOR complex 2 (mTORC2) controls hydrophobic motif phosphorylation and activation of serum- and glucocorticoid-induced protein kinase 1 (SGK1). Biochem J.

[29] Amato R, D'Antona L, Porciatti G, Agosti V, Menniti M, Rinaldo C (2009). Sgk1 activates MDM2-dependent p53 degradation and affects cell proliferation, survival, and differentiation. J Mol Med (Berl).

[30] Mayo LD, Donner DB (2001). A phosphatidylinositol 3-kinase/Akt pathway promotes translocation of Mdm2 from the cytoplasm to the nucleus. Proc Natl Acad Sci U S A.

[31] Murray JT, Campbell DG, Morrice N, Auld GC, Shpiro N, Marquez R (2004). Exploitation of KESTREL to identify NDRG family members as physiological substrates for SGK1 and GSK3. Biochem J.

[32] Murakami Y, Hosoi F, Izumi H, Maruyama Y, Ureshino H, Watari K (2010). Identification of sites subjected to serine/threonine phosphorylation by SGK1 affecting N-myc downstream-regulated gene 1 (NDRG1)/Cap43-dependent suppression of angiogenic CXC chemokine expression in human pancreatic cancer cells. Biochem Biophys Res Commun.

[33] Weiler M, Blaes J, Pusch S, Sahm F, Czabanka M, Luger S (2014). mTOR target NDRG1 confers MGMT-dependent resistance to alkylating chemotherapy. Proc Natl Acad Sci U S A.

[34] Tran H, Brunet A, Griffith EC, Greenberg ME (2003). The many forks in FOXO's road. Sci STKE.

[35] Liu W, Wang X, Liu Z, Wang Y, Yin B, Yu P (2017). SGK1 inhibition induces autophagy-dependent apoptosis via the mTOR-Foxo3a pathway. Br J Cancer.

[36] Mori S, Nada S, Kimura H, Tajima S, Takahashi Y, Kitamura A (2014). The mTOR pathway controls cell proliferation by regulating the FoxO3a transcription factor via SGK1 kinase. PLoS One.

[37] Zhang F, Virshup DM, Cheong JK (2018). Oncogenic RAS-induced CK1alpha drives nuclear FOXO proteolysis. Oncogene.

[38] Los M, Mozoluk M, Ferrari D, Stepczynska A, Stroh C, Renz A (2002). Activation and caspase-mediated inhibition of PARP: a molecular switch between fibroblast necrosis and apoptosis in death receptor signaling. Mol Biol Cell.

[39] Zhang CS, Hawley SA, Zong Y, Li M, Wang Z, Gray A (2017). Fructose-1,6-bisphosphate and aldolase mediate glucose sensing by AMPK. Nature..

[40] Prakasam G, Singh RK, Iqbal MA, Saini SK, Tiku AB, Bamezai RNK. Pyruvate kinase M knockdown-induced signaling via AMP-activated protein kinase promotes mitochondrial biogenesis, autophagy, and cancer cell survival. J Biol Chem. 2017;292(37):15561–76. 10.1074/jbc.M117.791343. 10.1074/jbc.M117.791343 PMC5602412 28778925

[41] Jeon SM, Chandel NS, Hay N (2012). AMPK regulates NADPH homeostasis to promote tumour cell survival during energy stress. Nature..

[42] Herzig S, Shaw RJ (2018). AMPK: guardian of metabolism and mitochondrial homeostasis. Nat Rev Mol Cell Biol.

[43] Bartolome A, Garcia-Aguilar A, Asahara SI, Kido Y, Guillen C, Pajvani UB, et al. MTORC1 regulates both general autophagy and Mitophagy induction after oxidative phosphorylation uncoupling. Mol Cell Biol. 2017. 10.1128/MCB.00441-17. 10.1128/MCB.00441-17 PMC5686580 28894028

[44] Golden EB, Cho HY, Hofman FM, Louie SG, Schonthal AH, Chen TC (2015). Quinoline-based antimalarial drugs: a novel class of autophagy inhibitors. Neurosurg Focus.

[45] Kimura T, Takabatake Y, Takahashi A, Isaka Y (2013). Chloroquine in cancer therapy: a double-edged sword of autophagy. Cancer Res.

[46] Rebecca VW, Amaravadi RK (2016). Emerging strategies to effectively target autophagy in cancer. Oncogene..

[47] Mulcahy Levy JM, Towers CG, Thorburn A (2017). Targeting autophagy in cancer. Nat Rev Cancer.

[48] Pascolo S (2016). Time to use a dose of chloroquine as an adjuvant to anti-cancer chemotherapies. Eur J Pharmacol.

[49] Tanenbaum L, Tuffanelli DL (1980). Antimalarial agents. Chloroquine, hydroxychloroquine, and quinacrine. Arch Dermatol.

[50] Yan Y, Xu Z, Dai S, Qian L, Sun L, Gong Z (2016). Targeting autophagy to sensitive glioma to temozolomide treatment. J Exp Clin Cancer Res.

[51] Li C, Liu Y, Liu H, Zhang W, Shen C, Cho K (2015). Impact of autophagy inhibition at different stages on cytotoxic effect of autophagy inducer in glioblastoma cells. Cell Physiol Biochem.

[52] Mulcahy Levy JM, Zahedi S, Griesinger AM, Morin A, Davies KD, Aisner DL, et al. Autophagy inhibition overcomes multiple mechanisms of resistance to BRAF inhibition in brain tumors. Elife. 2017;6. 10.7554/eLife.19671. 10.7554/eLife.19671 PMC5241115 28094001

[53] Klionsky DJ, Abdelmohsen K, Abe A, Abedin MJ, Abeliovich H, Acevedo Arozena A (2016). Guidelines for the use and interpretation of assays for monitoring autophagy (3rd edition). Autophagy..

[54] Pearson JRD, Regad T (2017). Targeting cellular pathways in glioblastoma multiforme. Signal Transduct Target Ther.

[55] Carracedo A, Ma L, Teruya-Feldstein J, Rojo F, Salmena L, Alimonti A (2008). Inhibition of mTORC1 leads to MAPK pathway activation through a PI3K-dependent feedback loop in human cancer. J Clin Invest.

[56] Serra V, Scaltriti M, Prudkin L, Eichhorn PJ, Ibrahim YH, Chandarlapaty S (2011). PI3K inhibition results in enhanced HER signaling and acquired ERK dependency in HER2-overexpressing breast cancer. Oncogene.

[57] Engelman JA, Chen L, Tan X, Crosby K, Guimaraes AR, Upadhyay R (2008). Effective use of PI3K and MEK inhibitors to treat mutant Kras G12D and PIK3CA H1047R murine lung cancers. Nat Med.

[58] Bedard PL, Tabernero J, Janku F, Wainberg ZA, Paz-Ares L, Vansteenkiste J (2015). A phase Ib dose-escalation study of the oral pan-PI3K inhibitor buparlisib (BKM120) in combination with the oral MEK1/2 inhibitor trametinib (GSK1120212) in patients with selected advanced solid tumors. Clin Cancer Res.

[59] De Luca A, Maiello MR, D'Alessio A, Pergameno M, Normanno N (2012). The RAS/RAF/MEK/ERK and the PI3K/AKT signalling pathways: role in cancer pathogenesis and implications for therapeutic approaches. Expert Opin Ther Tar.

[60] Buccarelli M, Marconi M, Pacioni S, De Pasqualis I, D'Alessandris QG, Martini M (2018). Inhibition of autophagy increases susceptibility of glioblastoma stem cells to temozolomide by igniting ferroptosis. Cell Death Dis.

[61] Mou Y, Wang J, Wu J, He D, Zhang C, Duan C (2019). Ferroptosis, a new form of cell death: opportunities and challenges in cancer. J Hematol Oncol.

[62] Wang Z, Liu P, Chen Q, Deng S, Liu X, Situ H (2016). Targeting AMPK signaling pathway to overcome drug resistance for Cancer therapy. Curr Drug Targets.

[63] Yoshida GJ (2015). Metabolic reprogramming: the emerging concept and associated therapeutic strategies. J Exp Clin Cancer Res.

[64] Vitale I, Manic G, Dandrea V, De Maria R (2015). Role of autophagy in the maintenance and function of cancer stem cells. Int J Dev Biol.

