# Supplementary material for: The kinase inhibitor SI113 induces autophagy and synergizes with quinacrine in hindering the growth of human glioblastoma multiforme cells
Source: J Exp Clin Cancer Res. 2019 May 17;38:202. doi: 10.1186/s13046-019-1212-1 (PMC6525441; doi:10.1186/s13046-019-1212-1)
Supplement: Supplementary file 1 — Table S1. Complete list of antibodies used for RPPA analysis and their main related information. (PDF 26 kb) [file 13046_2019_1212_MOESM1_ESM.pdf]

Supplementary Table S1. Complete list of antibodies used for RPPA analysis and their main related information. Antibodies higlighted in yellow have been selected for RPPA data analyses.

RPPA endpoint name	Item Name	Vendor	Catalog	Raised in	Recognizes	Antigen	Cell Signaling Technology Pathway
4E-BP1 pT37/46	Phospho-4E-BP1 (Thr37/46) Antibody	Cell Signaling Technology	9459	Rabbit	H M R Mk	EIF4EBP1	Translational Control
4E-BP1 pT70	Phospho-4E-BP1 (Thr70) Antibody	Cell Signaling Technology	9455	Rabbit	H M R Mk	EIF4EBP1	Translational Control
A-RAF pS299	Phospho-A-Raf (Ser299) Antibody	Cell Signaling Technology	4431	Rabbit	H M R	ARAF	MAP Kinase Signaling
ACACA pS79	Phospho-Acetyl-CoA Carboxylase (Ser79) Antibody	Cell Signaling Technology	3661	Rabbit	H M R Mk	ACACA|ACACB	Metabolism
AKT pS473	Phospho-Akt (Ser473) Antibody (disponibile anche come XP® Monoclonal	Cell Signaling Technology	9271	Rabbit	H M R Hm Dm B Dg Pg	AKT1| AKT2| AKT3	PI3K / Akt Signaling
AKT pT308	Phospho-Akt (Thr308) Antibody	Cell Signaling Technology	9275	Rabbit	H M R Hm	AKT1	PI3K / Akt Signaling
ALDH	Purified Mouse Anti-ALDH Clone 44/ALDH	BD	611194	Mouse	H	ALDH1A1	Metabolism
ALK	ALK (D5F3®) XP® Rabbit mAb	Cell Signaling Technology	3633	Rabbit mAb	H	ALK	RTK
ALK pY1586	Phospho-ALK (Tyr1586) Antibody	Cell Signaling Technology	3343	Rabbit	H	ALK	RTK
ALK pY1604	Phospho-ALK (Tyr1604) Antibody	Cell Signaling Technology	3341	Rabbit	H	ALK	RTK
AMPKα pS485	Phospho-AMPK-alpha-1 (Ser485) Antibody	Cell Signaling Technology	4184	Rabbit	H M R Mk	PRKAA1	Metabolism
AMPKα pT172	Phospho-AMPKa (Thr172) (40H9) Rabbit mAb	Cell Signaling Technology	2535	Rabbit	H M R Hm Mk Dm Sc	PRKAA1|PRKAA2	Metabolism
AMPKβ pS108	Phospho-AMPK-beta-1 (Ser108) Antibody	Cell Signaling Technology	4181	Rabbit	H M R Mk	PRKAB1|PRKAB2	Metabolism
Androgen-R pS650	Anti-Androgen Receptor (phospho S650) antibody	Abcam	ab47563	Rabbit	H M	AR	Nuclear Receptor Signaling CTR
Androgen-R pS81	Anti-phospho-Androgen Receptor (Ser81) Antibody	Millipore	07-1375	Rabbit	H	AR	Nuclear Receptor Signaling
AndrRec	Androgen Receptor Antibody	Cell Signaling Technology	3202	Rabbit	H	AR	Nuclear Receptor Signaling
ASK1 pS83	Phospho-ASK1 (Ser83) Antibody	Cell Signaling Technology	3761	Rabbit	H	MAP3K5	MAP Kinase Signaling
ATP-Citrate Lyase pS454	Phospho-ATP-Citrate Lyase (Ser454) Antibody	Cell Signaling Technology	4331	Rabbit	H M	ACLY	Metabolism
b-Catenin pS33-37/pT41	Phospho-beta-Catenin (Ser33/37/Thr41) Antibody	Cell Signaling Technology	9561	Rabbit	H M R Mk	CTNNB1	Developmental Biology
b-Catenin pT41/pS45	Phospho-beta-Catenin (Thr41/Ser45) Antibody	Cell Signaling Technology	9565	Rabbit	H M Mk	CTNNB1	Developmental Biology
BAD pS112	Phospho-Bad (Ser112) Antibody	Cell Signaling Technology	9291	Rabbit	H M R Mk	BAD	Apoptosis
BAD pS136	Phospho-Bad (Ser136) Antibody	Cell Signaling Technology	9295	Rabbit	M	BAD	Apoptosis
BAD pS155	Phospho-Bad (Ser155) Antibody	Cell Signaling Technology	9297	Rabbit	M	BAD	Apoptosis
c-ABL pT735	Phospho-c-Abl (Thr735) Antibody	Cell Signaling Technology	2864	Rabbit	H	ABL1|BCR/ABL	MAP Kinase Signaling
c-ABL pY245	Phospho-c-Abl (Tyr245) Antibody	Cell Signaling Technology	2861	Rabbit	H	ABL1 |EGFR|PDGFRA|P	MAP Kinase Signaling
c-KIT pY719	Phospho-c-Kit (Tyr719) Antibody	Cell Signaling Technology	3391	Rabbit	H M	KIT	RTK
c-Met	Met (D1C2) XP® Rabbit mAb	Cell Signaling Technology	8198	Rabbit mAb	H	MET	RTK
c-Met pY1234	Phospho-Met (Tyr1234/1235) Antibody (disponibile anche come XP® Mon	Cell Signaling Technology	3126	Rabbit	H M R Mk	MET	RTK
c-Myc pS62	Phospho-c-Myc (Ser62) (E1J4K) Rabbit mAb	Cell Signaling Technology	13748	Rabbit	H M R	MYC	Apoptosis
c-Myc pT58/pS62	Anti-phospho-c-Myc (Thr58/Ser62) Antibody	Millipore	04-217	Rabbit	H M	MYC	Apoptosis
C-RAF pS259	Phospho-c-Raf (Ser259) Antibody	Cell Signaling Technology	9421	Rabbit	H M R Mk X	RAF1	MAP Kinase Signaling
C-RAF pS338	Phospho-c-Raf (Ser338) (56A6) Rabbit mAb	Cell Signaling Technology	9427	Rabbit mAb	H M R Mk	RAF1	MAP Kinase Signaling
COX-2	Purified Mouse Anti-Cox-2 Clone 33/Cox-2	BD	610203	Mouse	H M C	PTGS2	Immunology and Inflammation
Cyclin B1	Cyclin B1 (V152) Mouse mAb	Cell Signaling Technology	4135	Mouse	H M	CCNB1	Cell Cycle / Checkpoint Control
Cyclin D1	Cyclin D1 (DCS6) Mouse mAb	Cell Signaling Technology	2926	Mouse	NA	CCND1	Cell Cycle / Checkpoint Control
E-Cadherin	E-Cadherin (24E10) Rabbit mAb	Cell Signaling Technology	3195	Rabbit	H M	CDH1	Adhesion/ECM
EGFR	EGF Receptor Antibody (disponibile anche come XP® Monoclonal Antibod	Cell Signaling Technology	2232	Rabbit	H M R Mk	EGFR	RTK
EGFR pY1045	Phospho-EGF Receptor (Tyr1045) Antibody	Cell Signaling Technology	2237	Rabbit	H R	EGFR	RTK
EGFR pY1068	Phospho-EGF Receptor (Tyr1068) Antibody (disponibile anche come XP®	Cell Signaling Technology	2234	Rabbit	H M R	EGFR	RTK
EGFR pY1148	Phospho-EGF Receptor (Tyr1148) Antibody	Cell Signaling Technology	4404	Rabbit	H Mk	EGFR	RTK
EGFR pY1173	Phospho-EGF Receptor (Tyr1173) (53A5) Rabbit mAb	Cell Signaling Technology	4407	Rabbit mAb	H M R	EGFR	RTK
EGFR pY845 MILK	Phospho-EGF Receptor (Tyr845) Antibody	Cell Signaling Technology	2231	Rabbit	H M R	EGFR	RTK
EGFR pY992	Phospho-EGF Receptor (Tyr992) Antibody	Cell Signaling Technology	2235	Rabbit	H M Mk	EGFR	RTK
ErbB2 pY1248	Phospho-HER2/ErbB2 (Tyr1248) Antibody	Cell Signaling Technology	2247	Rabbit	H M	ERBB2	RTK
ERG	ERG antibody [EPR3864(2)]	Epitomics/Abcam	5115-1/ab13326	Rabbit mAb	H M	ERG	N.A.
ERK1-2 pT202-pY204	Phospho-p44/42 MAPK (Erk1/2) (Thr202/Tyr204) Antibody (disponibile and	Cell Signaling Technology	9101	Rabbit	H M R Hm Mk Mi Dm Z	MAPK3|MAPK1	MAP Kinase Signaling
Estr-R alpha pS118	Phospho-Estrogen Receptor a (Ser118) (16J4) Mouse mAb	Cell Signaling Technology	2511	Mouse	H	ESR1	Nuclear Receptor Signaling
Fatty Acid Synthase	Fatty Acid Synthase Antibody	Cell Signaling Technology	3189	Rabbit	H M	FASN	Metabolism
Fodrin-a cl. D1185	Cleaved alpha-Fodrin (Asp1185) Antibody	Cell Signaling Technology	2121	Rabbit	H	SPTAN1	Apoptosis
FOXO1 pS256	Phospho-FoxO1 (Ser256) Antibody	Cell Signaling Technology	9461	Rabbit	H M R Mk	FOXO1|FOXO4	PI3K / Akt Signaling
FOXO1 pT24-FOXO3a pT32	Phospho-FoxO1 (Thr24)/FoxO3a (Thr32) Antibody	Cell Signaling Technology	9464	Rabbit	H M R Mk	FOXO1| FOXO4| FOXO3	PI3K / Akt Signaling
FSR2-a pY436	Phospho-FRS2-alpha (Tyr436) Antibody	Cell Signaling Technology	3861	Rabbit	H M	FRS2	MAP Kinase Signaling
GlucoCorticoid-R pS211	Phospho-Glucocorticoid Receptor (Ser211) Antibody	Cell Signaling Technology	4161	Rabbit	H M	NR3C1	Nuclear Receptor Signaling
Glutaminase	Glutaminase antibody [EP7212]	Abcam	ab156876	Rabbit mAb	H	GLS	Cytoskeletal Signaling
GSK3-α/β pS279	Phospho-GSK3 beta (Tyr216, Tyr279) Polyclonal Antibody	Biosource/Invitrogen	44-604G	Rabbit	H M R	GSK3A| GSK3B	PI3K / Akt Signaling
GSK3-α/β pS21-9	Phospho-GSK-3-alpha/beta (Ser21/9) Antibody	Cell Signaling Technology	9331	Rabbit	H M R Mk Z	GSK3A|GSK3B	PI3K / Akt Signaling
GSK3β pS9	Phospho-GSK-3-beta (Ser9) Antibody (disponibile anche come XP® Mono	Cell Signaling Technology	9336	Rabbit	H M R Mk	GSK3B	PI3K / Akt Signaling
H3-Histone pS10	Phospho-Histone H3 (Ser10) Antibody (disponibile anche come XP® Mon	Cell Signaling Technology	9701	Rabbit	H M R Mk Dm Sc	HIST1H3A	Chromatin Regulation / Nuclear Function
HER2 pY1248	ErbB2/Her2 [p Tyr1248] Antibody	Imgenex	IMG-90189-1	Rabbit	H M R	ERBB2	RTK
HER3 pY1289	Phospho-HER3/ErbB3 (Tyr1289) (21D3) Rabbit mAb	Cell Signaling Technology	4791	Rabbit mAb	H M	ERBB3	RTK
IGF1-R pY1131/Ins-R pY1146	Phospho-IGF-I Receptor beta (Tyr1131)/Insulin Receptor beta (Tyr1146) A	Cell Signaling Technology	3021	Rabbit	H M R	IGF1R	Metabolism
IGF1-R pY1135-6/Ins-R pY1150-1	Phospho-IGF-I Receptor ß (Tyr1135/1136)/Insulin Receptor ß (Tyr1150/11	Cell Signaling Technology	3024	Rabbit mAb	H M R	IGF1R	Metabolism
IRS-1 pS612	Phospho-IRS-1 (Ser612) Antibody	Cell Signaling Technology	2386	Rabbit	H M R	IRS1	Metabolism
LKB1 pS334	Phospho-LKB1 (Ser334) Antibody	Cell Signaling Technology	3055	Rabbit	H	STK11	Metabolism
LKB1 pS428	Phospho-LKB1 (Ser428) (C67A3) Rabbit mAb	Cell Signaling Technology	3482	Rabbit	H M R Mk	STK11	Metabolism
MDM2 pS166	Phospho-MDM2 (Ser166) Antibody	Cell Signaling Technology	3521	Rabbit	H M R	MDM2	Cell Cycle / Checkpoint Control
MEK1-2 pS217-21	Phospho-MEK1/2 (Ser217/221) Antibody	Cell Signaling Technology	9121	Rabbit	H M R Mk Sc	MAP2K1| MAP2K2	MAP Kinase Signaling
MKK4 pS80	Phospho-SEK1/MKK4 (Ser80) Antibody	Cell Signaling Technology	9155	Rabbit	H M R	MAP2K4	MAP Kinase Signaling
MSK1 pS360	Phospho-MSK1 (Ser360) Antibody	Cell Signaling Technology	9594	Rabbit	H	RPS6KA5	MAP Kinase Signaling

mTOR pS2448	Phospho-mTOR (Ser2448) Antibody (disponibile anche come XP® Monocl	Cell Signaling Technology	2971	Rabbit	H M R Mk	MTOR	PI3K / Akt Signaling
mTOR pS2481	Phospho-mTOR (Ser2481) Antibody	Cell Signaling Technology	2974	Rabbit	H M R Mk	MTOR	PI3K / Akt Signaling
NDRG1 pT346	Phospho-NDRG1 (Thr346) (D98G11) XP® Rabbit mAb	Cell Signaling Technology	5482	Rabbit	H M R Mk	NDRG1	Developmental Biology
NDRG1_pS330	Anti-NDRG1 (phospho S330) antibody [EPR5594]	Abcam	ab124713	Rabbit mAb	H M R	NDRG1	Developmental Biology CTR
p16_INK4A	p16 INK4A Antibody (DISCONTINUED)	Cell Signaling Technology	4824	Rabbit	NA	CDKN2A	Cell Cycle / Checkpoint Control
p21_Waf1/Cip1	p21 Waf1/Cip1 (12D1) Rabbit mAb	Cell Signaling Technology	2947	Rabbit	H Mk	CDKN1A	Cell Cycle / Checkpoint Control
p27_Kip1	Purified Mouse Anti-p27[Kip1] Clone 57/Kip1/p27	BD	610241	Mouse	H M R Dg C Fg	CDKN1B	Cell Cycle / Checkpoint Control
p38MAPK pT180-pY182	Phospho-p38 MAPK (Thr180/Tyr182) Antibody (disponibile anche come XF	Cell Signaling Technology	9211	Rabbit	H M R Mk Dm Pg Sc	MAPK14	MAP Kinase Signaling
p70S6K pT389	Phospho-p70 S6 Kinase (Thr389) Antibody	Cell Signaling Technology	9205	Rabbit	H M R Mk	RPS6KB1	PI3K / Akt Signaling
p70S6K pS371	Phospho-p70 S6 Kinase (Ser371) Antibody	Cell Signaling Technology	9208	Rabbit	H M R Mk	RPS6KB1	PI3K / Akt Signaling
p70S6K pT412	Anti-phospho-p70 S6 Kinase (Thr412) Antibody	Upstate/Millipore	07-018	Rabbit	H M	RPS6KB1	PI3K / Akt Signaling
p90RSK pS380	Phospho-p90RSK (Ser380) Antibody	Cell Signaling Technology	9341	Rabbit	H M R Mk	RPS6KA1	MAP Kinase Signaling
PARP cl. D214	Cleaved PARP (Asp214) Antibody (Human Specific)	Cell Signaling Technology	9541	Rabbit	H	PARP1	Apoptosis
PDGFR-α pY754	Phospho-PDGF Receptor alpha (Tyr754) (23B2) Rabbit mAb	Cell Signaling Technology	2992	Rabbit mAb	H M	PDGFRA	RTK
PDGFR-β pY716	Anti-phospho-PDGFRbeta (Tyr716) antibody	Upstate/Millipore	07-021	Rabbit	H M R	PDGFRB	RTK CTR
PDGFR-β pY751	Phospho-PDGF Receptor beta (Tyr751) Antibody	Cell Signaling Technology	3161	Rabbit	H M R	PDGFRB	RTK
PDK1 pS241	Phospho-PDK1 (Ser241) Antibody	Cell Signaling Technology	3061	Rabbit	H M R	PDPK1	PI3K / Akt Signaling
PLK1 pT210	Purified Mouse anti-PLK1 (pT210) Clone K50-483	BD	558400	Mouse	H (M)	PLK1	Cell Cycle / Checkpoint Control
PRAS40 pT246	Phospho-PRAS40 (Thr246) Polyclonal Antibody	Biosource/Invitrogen	44-1100G	Rabbit	H M R	AKT1S1	PI3K / Akt Signaling
ProgR pS190	Phospho-Progesterone Receptor (Ser190) Antibody	Cell Signaling Technology	3171	Rabbit	H	PGR	Nuclear Receptor Signaling
Pyruvate Carboxylase	PCB (D-9)	Santa Cruz Biotechnology	sc-365673	Mouse	H M R	PC	Metabolism
RanGAP pS428	Anti-phospho-RanGAP1 (pSer428) antibody	Sigma-Aldrich	R5280	Rabbit	H	RANGAP1	Ubiquitin and Ubiquitin-like proteins
S6 pS235-236	Phospho-S6 Ribosomal Protein (Ser235/236) (2F9)	Cell Signaling Technology	4856	Rabbit mAb	H M R Mk	RPS6	Translational Control
S6 pS240-244	Phospho-S6 Ribosomal Protein (Ser240/244)	Cell Signaling Technology	2215	Rabbit	H M R Mk Z	RPS6	Translational Control
SAP-JNK pT183-Y185	Phospho-SAPK/JNK (Thr183/Tyr185) Antibody	Cell Signaling Technology	9251	Rabbit	H M R Hm Mk Dm B Sc	MAPK8	MAP Kinase Signaling
SGK pS422	p-SGK (Ser 422)	Santa Cruz Biotechnology	sc-16745-R	Rabbit	H M R	SGK1	PI3K / Akt Signaling
SGK pS78	SGK (S78) (D36D11)	Cell Signaling Technology	5599	Rabbit mAb	H	SGK1	PI3K / Akt Signaling
SGK1 pT256	Phospho-SGK1 (Thr256) Polyclonal Antibody	Invitrogen	44-1260G	Rabbit	H M R	SGK1	PI3K / Akt Signaling
SGK3	SGK3 (D18D1)	Cell Signaling Technology	8156	Rabbit	H M R Mk	SGK3	PI3K / Akt Signaling
SHC pY317	Anti-phospho-SHC (Tyr317) Antibody	Upstate/Millipore	07-206	Rabbit	H M	SHC1	MAP Kinase Signaling
SMAD1-5-8 pS-S-S	Phospho-Smad1 (Ser463/465)/ Smad5 (Ser463/465)/ Smad8 (Ser426/428)	Cell Signaling Technology	9511	Rabbit	NA	SMAD1|SMAD5|SMAD8	Developmental Biology CTR
SMAD2 pS465-67	Phospho-Smad2 (Ser465/467) Antibody	Cell Signaling Technology	3101	Rabbit	NA	SMAD2	Developmental Biology
SRC Family pY416	Phospho-Src Family (Tyr416) Antibody	Cell Signaling Technology	2101	Rabbit	H M R	SRC	MAP Kinase Signaling
SRC pY527	Phospho-Src (Tyr527) Antibody	Cell Signaling Technology	2105	Rabbit	H M R	SRC	MAP Kinase Signaling
STAT1 pY701	Phospho-Stat1 (Tyr701) Antibody	Cell Signaling Technology	9171	Rabbit	NA	STAT1	Immunology and Inflammation
STAT3 pS727	Phospho-Stat3 (Ser727) Antibody	Cell Signaling Technology	9134	Rabbit	H M R	STAT3	Immunology and Inflammation
STAT3 pY705	Phospho-Stat3 (Tyr705) (D3A7) XP® Rabbit mAb	Cell Signaling Technology	9145S	Rabbit mAb	H M R Mk	STAT3	Immunology and Inflammation
STAT5 pY694	Phospho-Stat5 (Tyr694) Antibody	Cell Signaling Technology	9351	Rabbit	H M	STAT5	Immunology and Inflammation
TGF-beta	TGF-beta (56E4) Rabbit mAb	Cell Signaling Technology	3709	Rabbit	H	TGFB1|TGFB3	Developmental Biology
Tuberin pY1571	Phospho-Tuberin/TSC2 (Tyr1571) Antibody	Cell Signaling Technology	3614	Rabbit	M Mk	TSC2	PI3K / Akt Signaling
VEGFR2 pY1175	Phospho-VEGF Receptor 2 (Tyr1175) (19A10) Rabbit mAb	Cell Signaling Technology	2478	Rabbit mAb	H M	KDR	RTK
VEGFR2 pY951	Phospho-VEGF Receptor 2 (Tyr951) Antibody	Cell Signaling Technology	2471	Rabbit	H M	KDR	RTK
VEGFR2 pY996	Phospho-VEGF Receptor 2 (Tyr996) Antibody	Cell Signaling Technology	2474	Rabbit	H M	KDR	RTK
Vimentin	Vimentin (D21H3) XP® Rabbit mAb	Cell Signaling Technology	5741	Rabbit	H M R Mk	VIM	Cytoskeletal Signaling
WNT5a-b	Wnt5a/B (C27E8)	Cell Signaling Technology	2530	Rabbit mAb	H	WNT5A|WNT5B	Developmental Biology
