# Supplementary material for: The kinase inhibitor SI113 induces autophagy and synergizes with quinacrine in hindering the growth of human glioblastoma multiforme cells
Source: J Exp Clin Cancer Res. 2019 May 17;38:202. doi: 10.1186/s13046-019-1212-1 (PMC6525441; doi:10.1186/s13046-019-1212-1)
Supplement: Supplementary file 2 — Figure S1. Additional RPPA endpoints. (PDF 89474 kb) [file 13046_2019_1212_MOESM2_ESM.pdf]

Figure S1

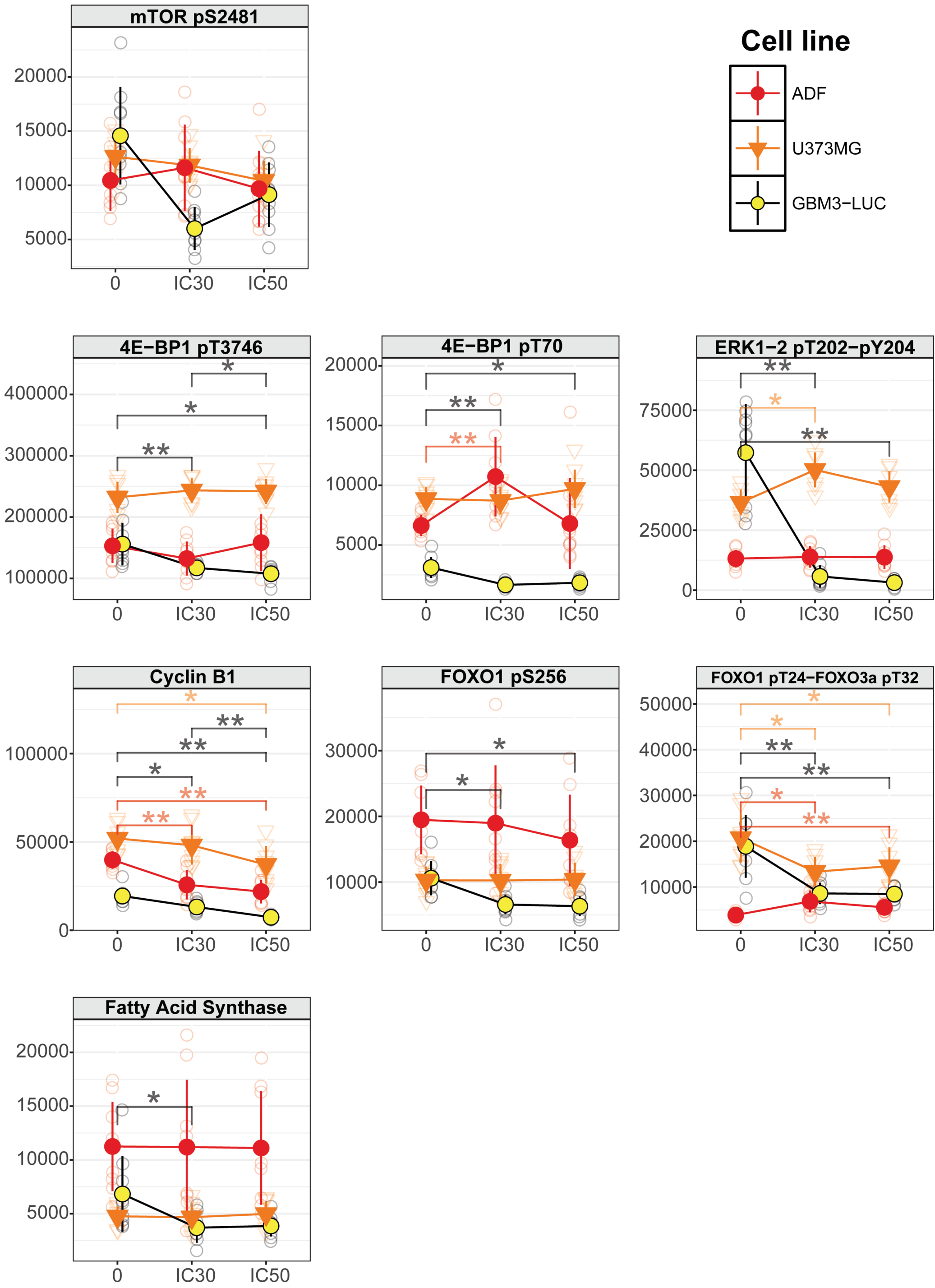

Figure S1. Additional RPPA endpoints. ADF, U373MG and GBM3-Luc cells were incubated with SI113 at the IC30 and IC50 concentrations specific for each cell line and then processed for RPPA analysis. When significant, statistical comparisons (Wilcoxon signed-rank test, FDR-adjusted p values) are reported on each individual plot. Statistical significance coding is described in the Materials and Methods section of the manuscript. RPPA plots refer to results obtained after 2 h (ERK1-2 pT202-pY204) or 8 h (mTOR pS2481, FOXO1 pS256, FOXO1 pT24-FOXO3a pT32 and Fatty Acid Synthase) of exposure to SI113.
