# Supplementary material for: The kinase inhibitor SI113 induces autophagy and synergizes with quinacrine in hindering the growth of human glioblastoma multiforme cells
Source: J Exp Clin Cancer Res. 2019 May 17;38:202. doi: 10.1186/s13046-019-1212-1 (PMC6525441; doi:10.1186/s13046-019-1212-1)
Supplement: Supplementary file 4 — Figure S2. Clonogenic Assay. (PDF 440 kb) [file 13046_2019_1212_MOESM4_ESM.pdf]

Figure S2

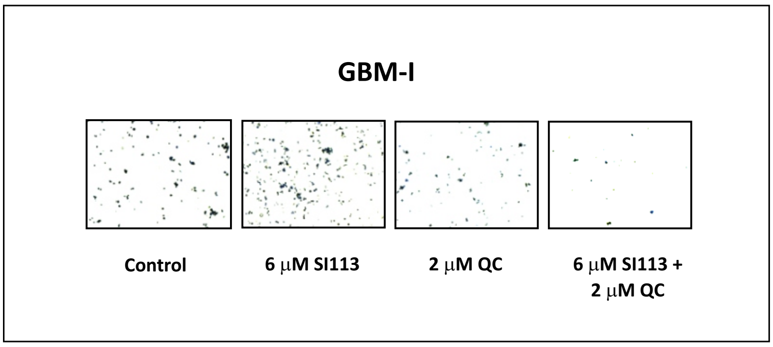

Figure S2. Clonogenic Assay. GBM-I cells were exposed to solvent(s) (Control), 6 μM SI113, 2 μM QC or their association for 48 h and then allowed to grow and form colonies for the subsequent 26 d. Then cells were all pelleted, fixed in 2% PFA, stained with 2% crystal violet, cytocentrifuged on a slide and
